# The phosphomimetic mutation of syndecan-4 binds and inhibits Tiam1 modulating Rac1 activity in PDZ interaction–dependent manner

**DOI:** 10.1371/journal.pone.0187094

**Published:** 2017-11-09

**Authors:** Aniko Keller-Pinter, Bettina Ughy, Monika Domoki, Aladar Pettko-Szandtner, Tamas Letoha, Jozsef Tovari, Jozsef Timar, Laszlo Szilak

**Affiliations:** 1 Department of Biochemistry, Faculty of General Medicine, University of Szeged, Szeged, Hungary; 2 Institute of Plant Biology, Biological Research Centre of the Hungarian Academy of Sciences, Szeged, Hungary; 3 Pharmacoidea Ltd., Szeged, Hungary; 4 Department of the Experimental Pharmacology, National Institute of Oncology, Budapest, Hungary; 5 II. Department of Pathology, Semmelweis University; MTA-SE Molecular Oncology Research Group, Budapest, Hungary; 6 Institute of Biology, Savaria Campus, Eötvös Lorand University, Szombathely, Hungary; 7 Szilak Laboratories Bioinformatics and Molecule-Design Ltd., Szeged, Hungary; Karolinska Institutet, SWEDEN

## Abstract

The small GTPases of the Rho family comprising RhoA, Rac1 and Cdc42 function as molecular switches controlling several essential biochemical pathways in eukaryotic cells. Their activity is cycling between an active GTP-bound and an inactive GDP-bound conformation. The exchange of GDP to GTP is catalyzed by guanine nucleotide exchange factors (GEFs). Here we report a novel regulatory mechanism of Rac1 activity, which is controlled by a phosphomimetic (Ser179Glu) mutant of syndecan-4 (SDC4). SDC4 is a ubiquitously expressed transmembrane, heparan sulfate proteoglycan. In this study we show that the Ser179Glu mutant binds strongly Tiam1, a Rac1-GEF reducing Rac1-GTP by 3-fold in MCF-7 breast adenocarcinoma cells. Mutational analysis unravels the PDZ interaction between SDC4 and Tiam1 is indispensable for the suppression of the Rac1 activity. Neither of the SDC4 interactions is effective alone to block the Rac1 activity, on the contrary, lack of either of interactions can increase the activity of Rac1, therefore the Rac1 activity is the resultant of the inhibitory and stimulatory effects. In addition, SDC4 can bind and tether RhoGDI1 (GDP-dissociation inhibitor 1) to the membrane. Expression of the phosphomimetic SDC4 results in the accumulation of the Rac1–RhoGDI1 complex. Co-immunoprecipitation assays (co-IP-s) reveal that SDC4 can form complexes with RhoGDI1. Together, the regulation of the basal activity of Rac1 is fine tuned and SDC4 is implicated in multiple ways.

## Introduction

The Rho GTPases, of which the best characterized are RhoA, Rac1 and Cdc42, are the major evolutionarily conserved regulators of the cytoskeleton, migration, cell polarity and differentiation [1,2]. They function as molecular switches cycling between an active GTP-bound and an inactive GDP-bound conformations. The GTP-loaded forms interact with the effector proteins inducing downstream signaling events. The activation cycle is primarily regulated by the guanine nucleotide exchange factors (GEFs), which catalyze the exchange of GDP for GTP. The intrinsic GTPase activity can be accelerated by GTPase-activating proteins (GAPs) facilitating their rapid inactivation [3]. The homeostasis and crosstalk among the members of the Rho-family is mediated by guanine nucleotide dissociation inhibitors (GDIs) keeping them in inactive GDP-bound form in the cytoplasm by association, shielding them from ubiquitylation and proteosomal degradation [4,5].

The release of the strongly bound GTPases from the RhoGDI1 complex is regulated mostly by specific phosphorylation of RhoGDI1; e.g. Rac1 is released upon phosphorylation of the Ser174 of RhoGDI1 by Pak1 (p21-activated kinase1) [6].

Pak1 was the first identified and the best characterized member of the PAK family, which is activated by Cdc42-GTP or Rac1-GTP [7]. PAKs have been implicated in the organization of the actin cytoskeleton, microtubule network, transcription regulation, death and survival signaling, and in the regulation of the cell cycle progression controlling G(2)/M transition and/or mitosis [8,9].

Tiam1, a typical member of the Dbl-like family of GEFs, activates the Rho-family GTPase Rac1 [10]. It is mainly involved in Rac1-dependent signaling pathways including cytoskeletal activities, cell polarity, endocytosis, membrane trafficking, cell migration, adhesion and invasion, cell growth and survival, metastasis and carcinogenesis [10,11]. The ubiquitously expressed Tiam1 serves not only as an enzyme but it acts as a scaffold protein harbouring several protein-protein interacting sites like the PH and PDZ domains [10]. The PDZ domains consist of approximately 90 amino acids and bind their recognition sequences of 4–10 amino acids at the C-terminus [12].

Syndecans constitute a family of four transmembrane heparan sulfate proteoglycans comprising a type II PDZ binding motif at their C-termini. Despite the conserved amino acid sequence of syndecans (EFYA) Tiam1 binds the different syndecans with altering affinities. It prefers SDC1 and SDC3 more than SDC2 or SDC4 [12]. Among the family members only SDC4 is expressed ubiquitously, involved directly in signal transduction, endocytic uptake and delivery of molecules and in the regulation of a variety of cellular processes such as migration, endocytosis, cytokinesis etc. [13,14]. The activity of SDC4 depends on the phosphorylation status of its cytoplasmic serine (Ser179 in human SDC4) [15–17]. The genetic knockout of SDC4 results in a constitutively high level of Rac1-GTP, which in turn manifested in mislocalization of the overabundant active Rac1 causing defect in cell migration and in planar cell polarity suggesting that SDC4 is involved in maintaining a low basal activity of Rac1 [18,19]. Overexpression of the non-phosphorylatable or the phosphomimetic mutants of SDC4 led to cytokinesis failures [17,19]. Previous studies showed that FGF2 stimulation or wound healing resulted in elevated activity of Rac1, involved in the PKC alpha pathway [20,21].

Here we report that Tiam1 and SDC4 were pulled down together in SDC4 phosphorylation- and PDZ-dependent manner controlling the bound Rac1 in the Tiam1 complex. The basal level of the Rac1 activity was regulated thereby. Further, we examined the role of RhoGDI1 in the Rac1 activation and showed that RhoGDI1 was pulled down with SDC4 independently from the mutations, and RhoGDI1—Rac1 was accumulated in the cells expressing the phosphomimetic mutant SDC4.

## Results

### SDC4 can bind Tiam1 mediating the interaction of Rac1—Tiam1

**Fig 1:**
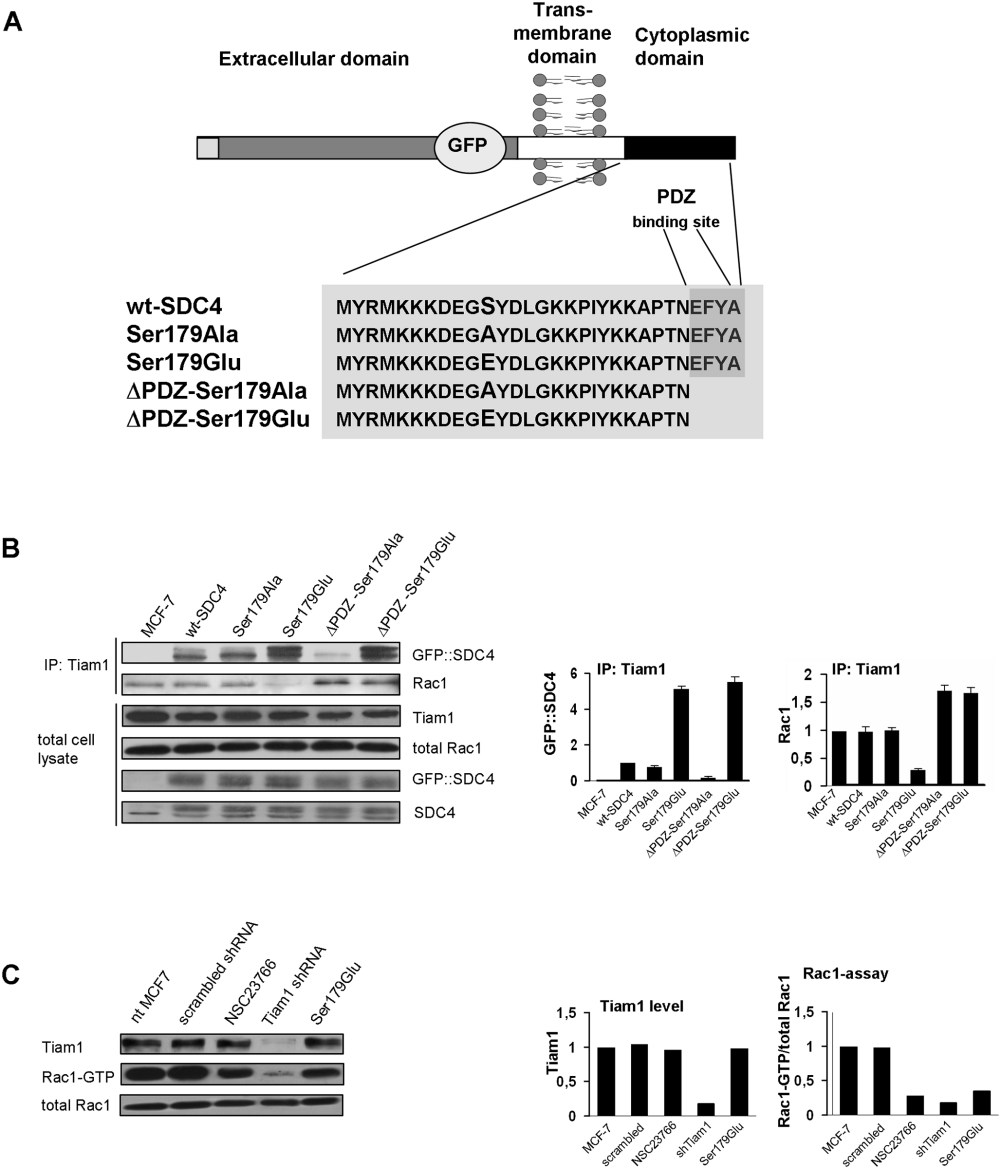
SDC4 controls the activity of Tiam1. (A) Schematic representation of SDC4 clones. The Ser179 of the cytoplasmic domain of SDC4 was mutated to non-phosphorylatable Ala (Ser179Ala) or, phosphomimetic Glu (Ser179Glu), respectively. The type II PDZ binding site was deleted in the Ser179 mutants generating ΔPDZ constructs. (B) Co-IPs were carried out with anti-Tiam1 antibody from the lysates of the different SDC4 lines and the subsequent immunoblot was probed with anti-GFP-HRP (GFP::SDC4) or anti-Rac1 antisera, respectively. Tiam1, Rac1 and GFP::SDC4 were used as loading controls. The strongest interactions were detected in the Ser179Glu and ΔPDZ-Ser179Glu cell lines. The Rac1 was greatly reduced in Tiam1 co-IP in the lysates of Ser179Glu cells only. The bar diagrams show the relative amount of the ectopic SDC4 normalized to wt-SDC4 cells, and pulled-down Rac1 normalized to MCF-7 cells. The results of panel B are representatives at least 3 independent experiments; data are reported as mean ± SEM (n = 3). (C) The protein level of Tiam1 and the Rac1 activity was monitored in non-treated MCF7 cells and upon transfection with scrambled sequence of Tiam1 shRNA construct, administration of NSC23766, transfection with Tiam1 shRNA, and in Ser179Glu SDC4 cells, respectively. Rac1 was used as loading control. Bar diagrams represent the quantification of the level of Tiam1 protein normalized to the nt MCF-7 cells and the ratio of Rac1-GTP and the endogenous Rac1 normalized to MCF-7 cells. The Tiam1 shRNA reduced the expression of Tiam1, and the Rac1 activity, too. The administration of NSC23766 and the expression of Ser179Glu SDC4 decreased the activity of Rac1 solely, the level of Tiam1 was unchanged. The use of the scrambled sequence of Tiam1 shRNA did not influence the expression level of Tiam1 and the Rac1-GTP either.

Tiam1 is a ubiquitously expressed Rac1-GEF comprising a PDZ domain [22,23], and it was demonstrated to bind the PDZ binding site of syndecans [23–25]. To gain insight into the SDC4-mediated signaling we introduced several mutations into SDC4. Since in the human SDC4 the Ser179 functions as a phosphorylation-dependent switch [16] thus we generated non-phosphorylatable Ser179Ala, and phosphomimetic Ser179Glu SDC4 constructs. Further, the interaction between Tiam1 and syndecans was characterized mainly as PDZ binding so double mutants were used: ΔPDZ-Ser179Ala and ΔPDZ-Ser179Glu SDC4. The mutants were compared to the wild-type (wt) SDC4. Green fluorescent protein (GFP) was introduced into the extracellular domain of SDC4 in order to avoid the interference with the cytoplasmic domain (Fig 1A).

We investigated the interaction between SDC4 and Tiam1 with co-immunoprecipitation (co-IP) experiments using anti-Tiam1 antibody, at the same time monitored the connection of Tiam1 and Rac1 (Fig 1B). SDC4 and Rac1 were tested in the immunoprecipitate of Tiam1. We found that Tiam1 pulled down the phosphomimetic SDC4 mutants (Ser179Glu and ΔPDZ-Ser179Glu) significantly more strongly, than the other SDC4 forms, regardless of the presence of the PDZ-binding site. On the other hand the interaction between Tiam1 and the PDZ binding site deleted, non-phosphorylatable form (ΔPDZ-Ser179Ala) was very weak (Fig 1B/1st slab).

At the same time the interaction of Rac1 and Tiam1 was investigated in the same immunoprecipitate (Fig 1B) following the standard method [26]. In Tiam1 immunoprecipitate of nt, wt and Ser179Ala MCF-7 cells the amount of Rac1 was commeasurable, more abundant Rac1 was observed in the cell lines expressing the PDZ binding site deletion SDC4 mutants, and importantly Rac1 was hardly observed in the cells expressing the Ser179Glu mutant SDC4 (Fig 1B/2nd slab). The expression levels of Tiam1, Rac1 and SDC4 constructs were unchanged (Fig 1B).

**Fig 2:**
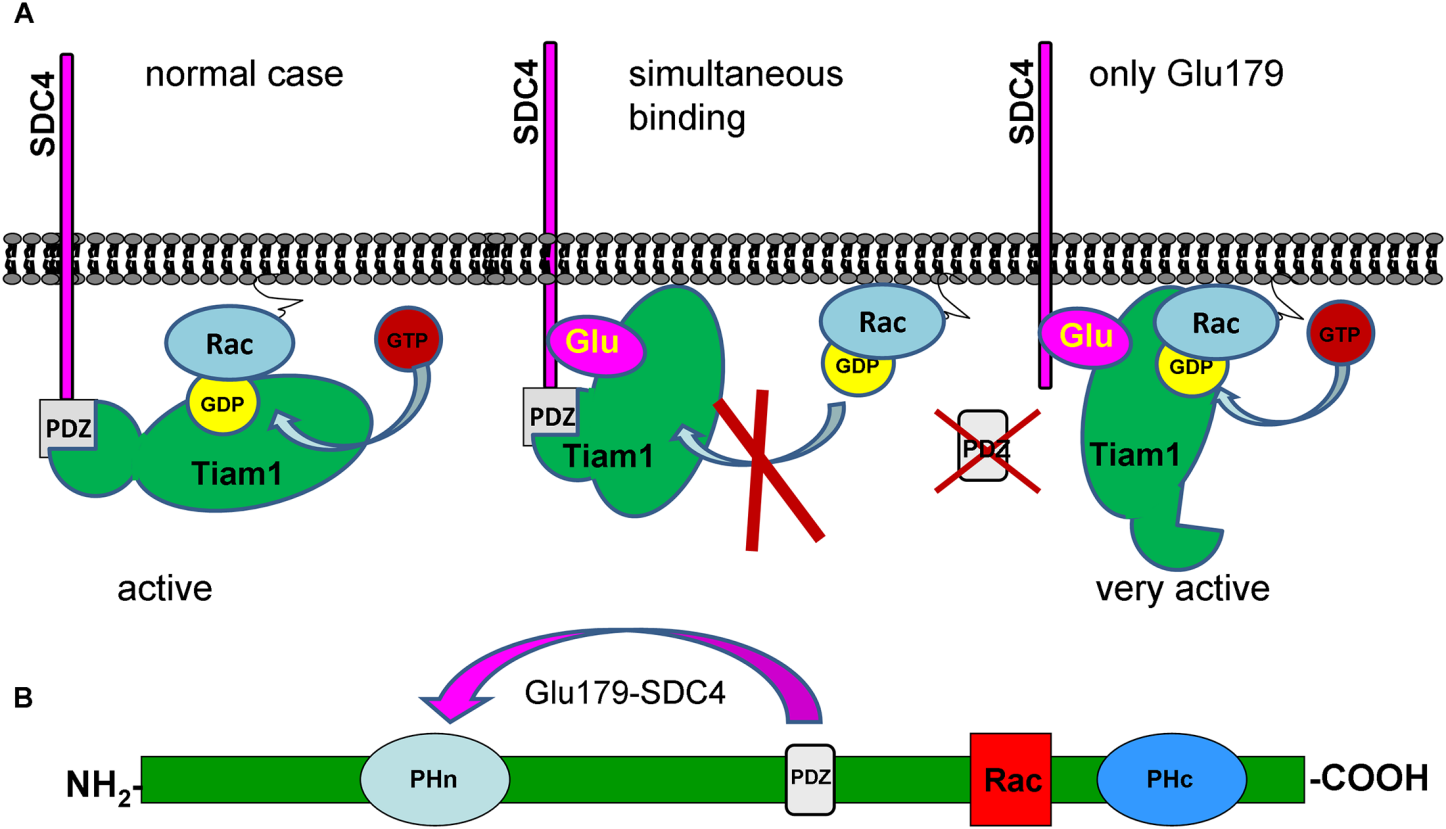
Schematic representation of the assumed mechanism of the inhibition of Tiam1 by phosphomimetic SDC4. (A) The only connection of SDC4 to Tiam1 via PDZ binding site or Glu^179^ does not interfere with Tiam1 activity; however the simultaneous interactions block the enzyme activity. (B) Structure of Tiam1 contains two pleckstrin homology domains (PHn and PHc). Rac1 binding site is located between the PHn and PHc domains. In pull down experiment PH domain was identified as interaction site, and PHn was shown to regulate binding of GTPases [10] thus we suppose that phosphomimetic SDC4 can interact with the PDZ and PHn domains simultaneously to exclude Rac1.

Taken together, the endogenous Tiam1 level was not changed by the expressions of the different SDC4 constructs but the pulled down SDC4 and Rac1 were varied in the Tiam1 precipitates meaning that the association of Rac1 and Tiam1 was controlled by SDC4 in the different cell lines. Since the interaction of Ser179Glu SDC4 and Tiam1 was the strongest and the bound Rac1 was reduced in the Tiam1 complex, we concluded that the phosphomimetic SDC4 with PDZ binding site restricted the interaction between Tiam1 and Rac1. The deletion of the PDZ binding site (ΔPDZ-Ser179Glu) enhanced the Rac1binding to Tiam1. Therefore to block the enzymatic function of Tiam1 simultaneous interactions of the phosphomimetic mutation and PDZ binding site of SDC4 were needed. We suggested a model, how phosphomimetic SDC4 could restrict the binding of Rac1 to Tiam1 (Fig 2). Most probably the PDZ domain and the N-terminal pleckstrin homology (PH) domain can interact with Ser179Glu mutation of SDC4 (Fig 2B). With affinity-based proteomics PH domain was detected in ΔPDZ-Ser179Glu cells with 7/7/28.3% and in Ser179Glu cells with 6/8/25.1% frequency (number of unique peptide/ peptide count/ coverage, respectively). (In an independent repeat the values were 2/2/8.7% and 3/3/11.3%, in ΔPDZ-Ser179Glu, or in Ser179Glu cells, respectively.)

Next we investigated how Tiam1 contributed to the Rac1-GTP level of MCF7 cells. To do so, the level of Rac1-GTP was characterized in different conditions and compared to that of the Ser179Glu SDC4 cells. The cells were transfected transiently by Tiam1 shRNA construct, and with its scrambled sequence or the media was supplemented with NSC23766 (Fig 1C). The NSC23766 can bind Rac1 preventing Rac1 activation by TrioN and Tiam1 without affecting Cdc42 or RhoA activation [27]. We found that the level of Tiam1 in the Tiam1 shRNA expressing cells was decreased by ~90%; in accordance with the reduction of the activity of Rac1 (Fig 1C). The NSC23766 treatment decreased the Rac1-GTP by ~70%, while the expression of the Ser179Glu SDC4 reduced it by ~60% comparing to the nt MCF-7 cells. The transfection of MCF7 with scrambled sequence of Tiam1 shRNA vector changed neither the amount of Tiam1, nor that of the active Rac1 (Fig 1C).

### The activity of Rac1 is modulated by phosphomimetic SDC4

**Fig 3:**
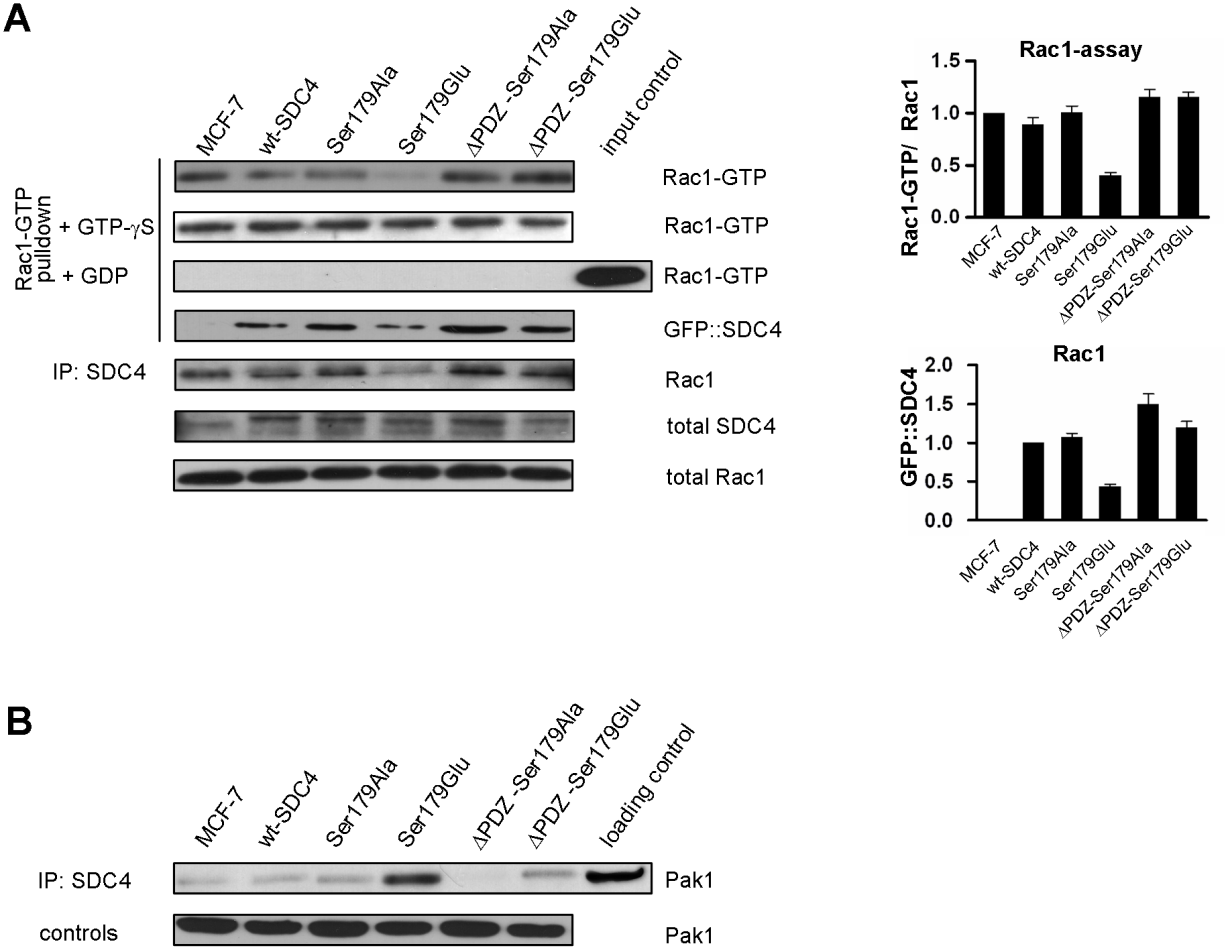
The activity of Rac1 is modulated by SDC4. (A) The level of active Rac1-GTP was monitored in MCF-7 cells expressing different SDC4 constructs by pull-down assay employing CRIB domain of Pak1. The Ser179Glu cells contained the least Rac1-GTP among the cell lines. Administration of non-hydrolysable GTP-γS equalized the amount of pulled-down Rac1, while the high access of GDP eliminated it. Rac1 and GFP were used as loading control. Bar diagrams represent the quantification of the ratio of Rac1-GTP and the endogenous Rac1 normalized to MCF-7 cells. According to this the Rac1 activity of the control cells was reduced 3-fold in the Ser179Glu cells and was increased by 15% in the ΔPDZ cells. In the 4th slab SDC4 was probed on the CRIB-based Rac1 assay. In the 5th panel Rac1 was tested in GFP co-IPs. The pattern is similar to that of Rac1 received in the Rac-assay. MCF-7 line served as a negative control. The results of panel A are representatives of at least 5 independent experiments; data are reported as mean ± SEM (n = 5). (B) Pak1 kinase was probed in the SDC4 co-IPs. The most abundant Pak1 was detected in the Ser179Glu mutant cells. Pak1 was used as loading control.

The level of Rac1-GTP was characterized in cells expressing the different SDC4 mutants with a specific pull-down assay using the Cdc42/Rac1 interactive binding (CRIB) domain of Pak1 (Fig 3). The activity of Rac1 varied in the different cell lines. Surprisingly, it was reduced about to one third (0.34±0.02) in the Ser179Glu cells, and it was increased (by ~15%) in cells expressing PDZ binding site deletion mutants comparing to those of nt-, wt—and Ser179Ala cells (Fig 3A/1st slab). As control experiments the cell lysates of the different clones were saturated with non-degradable GTP-γS or GDP to exclude the artefacts. The stable GTP-γS equalized the amount of the detected Rac1-GTP, while the high access of GDP totally eliminated the interactions (Fig 3A).

We were curious whether SDC4 could be detected in the Rac-assay, so SDC4 was tested with anti-GFP antibody. SDC4 was pulled down by CRIB domain-based assay with similar pattern that was observed in the case of Rac1 (Fig 3A/ 4th slab). Next, we executed a co-IP experiment with anti-GFP antibody to precipitate SDC4 proteins and monitored Rac1 in the precipitate. Rac1 was detected in the precipitate with similar pattern that was seen in the Rac1-assay (Fig 3A/5th slab).

Because of Pak1 kinase is involved in the regulation of Rac1 activity, we investigated the interaction of SDC4 with Pak1 kinase. To do so the presence of Pak1 was tested in SDC4 co-IP (Fig 3B). Indeed, Pak1 was observed in the immunoprecipitate of the different cell lines. However, the most abundant Pak1 was observed in the immunoprecipitates of the Ser179Glu SDC4 expressing cell lines (Fig 3B). The nature of the interactions of SDC4 with Rac1-CRIB complex or with PAK1 is totally inverse, therefore we do not think that SDC4 could bind Rac1 on the effector site.

### PKC alpha activity did not influence the basal level of Rac1-GTP in MCF-7 cells

Previous studies showed that PKC alpha could contribute to the activation of Rac1 [18–21] through the RhoG-ELMO-DOCK180 complex [30], therefore we tested, how PKC alpha could be implicated in the setting of the basal level of Rac1-GTP. The mutation of cytoplasmic serine of SDC4 was characterized and the Glu mutation reduced the super activation of PKC alpha [16]. Further Gö6976, a specific PKC alpha inhibitor was administered to the exponentially growing MCF-7 cell lines and the level of the Rac1-GTP was monitored. According to our data the administration of Gö6976 did not change the Rac1 activity in MCF7 cells, further it did not influence the observed activation pattern of Rac1 (S1A Fig). Thus the concentration of Rac1-GTP of the control cells was reduced to approximately 30% in the Ser179Glu cells. We can state, that the PKC alpha mediated pathway is probably dispensable for setting the basal activity of Rac1 in MCF-7 cells.

### The SDC4—RhoGDI1 interaction in the Rac1 activation cycle

**Fig 4:**
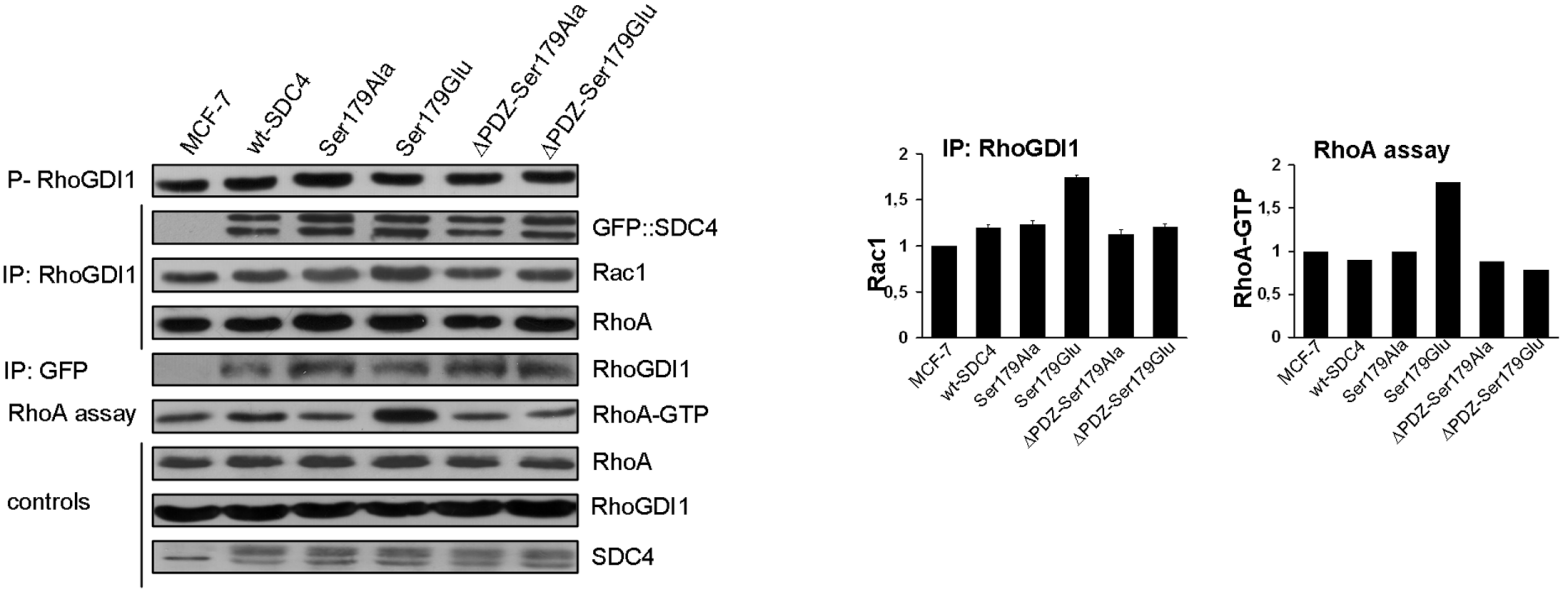
SDC4 associates with RhoGDI1 and Pak1. (A) The phosphorylation level of the regulatory Ser174 of RhoGDI1 was tested and no significant differences were found in the indicated cell lines (1st slab). RhoGDI1 co-IPs were performed to test direct interactions with GFP::SDC4, Rac1, or RhoA in the different cell lines (2nd, 3rd and 4th slabs, respectively), and RhoGDI1 was monitored in GFP co-IPs (5th slab). RhoGDI1 and SDC4 were detected mutually in their immunoprecipitates. Importantly, the lack of PDZ binding site did not influence the interaction between RhoGDI1 and SDC4. In the RhoGDI1 co-IPs elevated Rac1 was observed in the Ser179Glu line (3rd slab; 1st bar diagram, right side). At the same time the level of the pulled down of RhoA was uniform (4th slab). RhoA-GTP was monitored in the different cell lines with rotekin-based assay. The active RhoA was increased approximately 2-fold in the Ser179Glu line compared to nt MCF7 (6th slab, and 2nd bar diagram, right side). RhoA, RhoGDI1 and SDC4 were used as loading controls. Bar diagrams represent Rac1 pulled-down by anti-RhoGDI1, and active RhoA normalized to the nt MCF7 cells. These results are representatives of 3 independent experiments; data are reported as mean ±SEM (n = 3).

Blocking an intermediate step of a cyclic metabolic pathway arrests the cycle leading to accumulation of the downstream and depletion of the upstream intermediates. Therefore we investigated whether SDC4 had any effect on the accumulation of RhoGDI1-Rac1, which might influence the regulation of the activity of Rac1. The phosphorylation of the regulatory Ser174 of RhoGDI1 [6] was monitored by immunoassay and the RhoGDI1–Rac1 interaction by co-IP experiments (Fig 4). We could not find significant difference in the level of the phosphorylation of RhoGDI1 in the different cell lines (Fig 4/1st slab).

SDC4 was suggested to tether RhoGDI1 to the membrane via PDZ interaction of synectin establishing SDC4-synectin-RhoGDI1 complex. However in that study direct interaction between SDC4 and RhoGDI1 was failed to be investigated [20]. Thus, we examined the interaction between SDC4 and RhoGDI1 by co-IP experiments using antibodies against GFP::SDC4 and RhoGDI1. Both proteins were mutually detected abundantly in the immunoprecipitates of RhoGDI1 or GFP (GFP::SDC4) (Fig 4/2nd and 5th slabs) independently from the presence of PDZ binding site, therefore the interaction of SDC4 with RhoGDI1 is not dependent on PDZ interaction.

Since, the phosphorylation of RhoGDI1 was even (Fig 4/1st slab) and the expression level of RhoGDI1 was also unchanged (Fig 4/8th slab) in the examined cell lines we were interested in the amount of Rac1 in the RhoGDI1–Rac1 complex. To study this Rac1 was investigated in the co-IP of RhoGDI1 of the different cell lines (Fig 4/3rd slab). Increased amount of Rac1 was observed in the Ser179Glu cells with an extent of approximately 50% (Fig 4/3rd slab, and 1st bar diagram). The interaction of RhoGDI1 and Ser179Glu was confirmed by proteomic analysis, where RhoGDI1 was detected in ΔPDZ-Ser179Glu cells with 3/3/23% and in Ser179Glu cells with 3/4/31.4% frequency (number of unique peptide/ peptide count/ coverage, respectively). (In independent repeat the values were 1/1/16.2% and 1/2/16.2%, in ΔPDZ-Ser179Glu, or in Ser179Glu cells, respectively.)

Taken together, the phosphorylation of Ser174 of RhoGDI1 was unchanged in the cells expressing different SDC4 mutants; however the stability of the Rac1-RhoGDI1 complex was increased in the cells expressing the Ser179Glu mutation. If there is a given pool of RhoGDI and the members of the Rho-GTPase family members compete for GDI binding to avoid the degradation [4,5] the access of Rac1 should displace other GTPases in RhoGDI1 complex. Thus we investigated if RhoA was affected by the increased Rac1-RhoGDI1. The amount of the pulled down RhoA in the RhoGDI1 IP was constant (Fig 4/5th slab), therefore the pool of RhoA—RhoGDI1 complex was not disturbed.

Because of the activity of RhoA is antagonistic with that of Rac1 [4] and the activity of Rac1 alternated we studied the RhoA activity with Rhotekin-based assay whether there was any effect of the decrement of the Rac1 activity on the RhoA activity (Fig 4/6th slab). Indeed, in the Ser179Glu cell line, where the Rac1-GTP was reduced the activity of RhoA was the most intensive; it was elevated approximately 2 fold (Fig 4/2nd bar diagram).

## Discussion

The activity of Rho family GTPases is highly tuned process. Any perturbation in their activity leads to the failure in polarization, migration, cell proliferation [1,2,31,32]. SDC4 was implicated in maintaining basal Rac1 activation [18,19], however, the state-of-the-art reported on the role of SDC4 in the Rac1 activation under stress conditions [20]. Our results unravelled a novel regulatory mechanism of the basal Rac1 activity that was mediated by SDC4 in a phosphorylation- and PDZ interaction-dependent manner in MCF-7 breast adenocarcinoma cells. The phosphomimetic SDC4 mutant could associate to Tiam1 the major Rac1-GEF of MCF-7 cells via at least two positions: at the Ser179Glu and through the PDZ binding site. Simultaneous bindings at the two positions reduced the Rac1 activity ~3-fold. Either interaction alone increased the activity of Rac1. Further, we observed that SDC4 could interact directly with RhoGDI1 increasing the stability of the Rac-RhoGDI1 complex.

### The phosphomimetic SDC4 has high affinity for Tiam1

Earlier the PDZ interaction of Tiam1 and syndecans was studied and it was shown that Tiam1 binds syndecans with different strength; SDC1 or SDC3 were bound 15-fold stronger than SDC2 or SDC4 [12,25]. However, our mutational experiments revealed that the phosphomimetic SDC4 bound Tiam1 approximately ten times better than the non-phosphorylated one, meaning that SDC4 phosphorylation enhanced the affinity for Tiam1. At the same time the presence of the PDZ domain did not change the affinity of the phosphomimetic SDC4 for Tiam1 significantly, meaning that the PDZ binding is weak (Fig 1). Further, proteomic analysis of Ser179Glu SDC4 confirmed an interaction of Tiam1 and Ser179Glu SDC4, through the PH domain of Tiam1.

Studying the Tiam1 –Rac1 interaction we found that the amount of Rac1 pulled down by Tiam1 was reduced greatly in the cells expressing the phosphomimetic SDC4. On the contrary, the deletion of PDZ binding site of SDC4 increased the pulled down Rac1 by Tiam1 and consequently the Rac1 activity by 20%. Therefore we surmise that the only interaction of the phosphomimetic SDC4 might relief the auto inhibitory conformation of Tiam1 by binding its N-terminal region. The simultaneous interactions of PDZ and the 179Glu are required to maintain the inactive conformation of Tiam1 [28,29,11] (Fig 2).

It has already been suggested that SDC4 is necessary to suppress Rac1 activity; however the suggested regulatory mechanism was characterized in stress conditions [19–21]. Here we unravelled that the Ser179Glu SDC4 can down regulate the basal activation of Rac1. The inhibitory effect of the phosphomimetic mutant was comparable to that of NSC23766, although NSC23766 was a slightly better Rac1-GTP inhibitor (by ~ 10%) than Ser179Glu SDC4. It should be noted that NSC23766 has wider specificity inhibiting Tiam1 and TrioN [27]. There are some further consequences of the depressed activity of Rac1 e.g. the activity of RhoA was increased, because the Rac1 and RhoA activity is mutually exclusive [4,31].

### Interaction of SDC4 and RhoGDI1

The Rac1 activation circle can also be driven by the release of Rac1 from the RhoGDI1-Rac1 complex that depends on the phosphorylation of Ser174 of RhoGDI1 by Pak1 [6]. The phosphorylation of Ser174 of RhoGDI1 was tested and found constant in the different cell lines. Rac1 was investigated in the RhoGDI1 complex and we observed that it was elevated slightly in the Ser179Glu mutant cells. Studies emphasized that there is a given pool of RhoGDI and the members of the Rho-GTPases should compete for GDI binding otherwise they will be degraded, thus other Rho-GTPases should be displaced by the excess of Rac1 [4,5]. We checked RhoA in the RhoGDI1 complex, and the pulled down RhoA was unaffected in the different cell lines.

The direct interaction of SDC4 and RhoGDI1 was investigated in co-IP experiments. They could pull down each other conversely and this interaction independent of the PDZ binding site (Fig 4). The interaction was confirmed with a proteomic analysis with Ser179Glu SDC4, where this interaction was detected. These together argue the importance of the PDZ interaction in the RhoGDI1 binding, because earlier works suggested the involvement of PDZ interaction in RhoGDI1–synectin-SDC4 complex. Most probably SDC4 can tether RhoGDI1 alone to the membrane establishing a SDC4 -RhoGDI1-synectin complex [20].

The accumulation of RhoGDI1-Rac1 complex in the Ser179Glu cells is very interesting. It definitely means that PDZ binding is necessary to the release of Rac1 from RhoGDI1, therefore other protein(s) should be involved in the Rac1 release that might be synectin.

In the cells the total amount of Rac1 was constant, at the same time the active Rac1 was reduced, and the amount of the Rac1-RhoGDI1 complex was elevated in the Ser179Glu cell line. We tested RhoA in complex of RhoGDI1, but it was not affected by the access of Rac1. However, the active RhoA was increased approximately two-fold that is most probably due to the reciprocal balance of the RhoA—Rac1 activity.

We could not find any evidence for the implication of PKC alpha in the regulation of the basal activity of Rac1 in MCF7 cells, although the indispensable role of PKC alpha was emphasized in the regulation of the activity of Rac1 in some stress conditions [18,20].

Activation of Tiam1 and consequently Rac1 was reported in T cells upon TCR-induced activation of LFA-1 (α_L_β_2_ integrin heterodimer). Down-regulation of Tiam1 inhibited Rac1 activity, cell adhesion, and migration [33]. This is a good example that the Rac1 activity is driven by Tiam1 mostly and is regulated dynamically by outside-in signals.

### SDC4 forms a complex with Pak1

Since the amount of Rac1-GDP in the membrane is regulated with the phosphorylation of Ser174 of RhoGDI1 by Pak1 [6], we investigated whether the expression of the SDC4 clones influence the phosphorylation status of RhoGDI1, because SDC4 pulled down RhoGDI1 and Pak1, as well. We did not find any evidences that SDC4 would be implicated in the regulatory phosphorylation of RhoGDI1.

Pak1 was among the firsts to be identified as Rac1/Cdc42 effector kinase [8]. Spatiotemporal regulation of Pak1 activity is essential in cytoskeleton dynamics, cell migration, polarization, cell cycle progression as well as in cell survival. Several adaptor proteins e.g. GIT1, Nck, Grb2 can bind Pak1 recruiting its activity to the membrane [7,8,34,35]. Here we showed that SDC4 can bind Pak1 and the phosphomimetic SDC4 had the greatest affinity for it. Most probably the activity of Pak1 can be regulated at least spatio-temporally in a SDC4 phosphorylation manner. The interaction of SDC4 and Pak1 has been already assumed; however direct interaction was not shown between them [36]. Here we could demonstrate first an interaction between SDC4 and Pak1. It could be intriguing a Tiam1-SDC4-Pak1 signaling complex that should be elucidated in further experiments.

**Fig 5:**
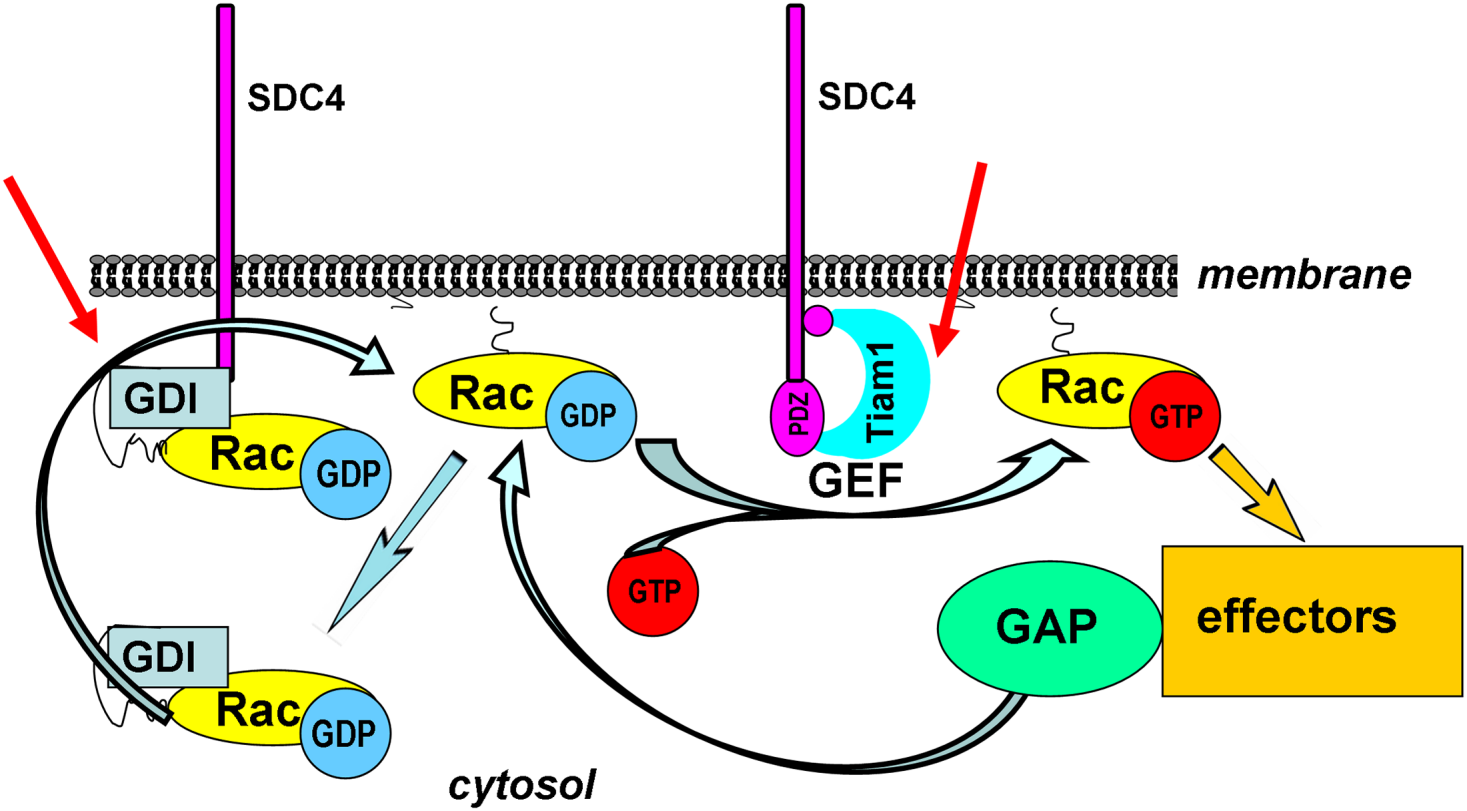
Schematic representation of the assumed mechanism of the regulation of Rac1 activity by SDC4. The activation cycle of Rac1 is regulated by several factors. GEFs catalyze the exchange of GDP for GTP. The intrinsic GTPase activity can be accelerated by GAPs. The GDIs can extract Rac1 from the membrane keeping it mostly in GDP-bound form in the cytosol. The red arrows show the potential regulatory sites of the phosphomimetic SDC4, 1) inhibiting Tiam1 a Rac1-GEF and 2) inducing the accumulation of the cytosolic Rac1-RhoGDI1 complex.

Taken together, we concluded that SDC4 might be involved in several ways in the activation cycle by 1) blocking a Rac1-GEF complex and 2) interfering with RhoGDI1–Rac1 complex to promote the release of Rac1 (Fig 5).

## Materials and methods

### Cell culture

MCF-7 human breast adenocarcinoma cells were obtained from ATCC, cultured in DMEM:F12 (Sigma-Aldrich) containing penicillin-streptomycin-amphoterycin B (Lonza, Basel, Switzerland) and supplemented with 10% fetal bovine serum (FBS) (Life Technologies/Gibco), unless otherwise stated. The stably transfected cell lines were propagated in DMEM:F12 medium supplemented with 10% FBS and 200 μg/ml G418 (Cambrex).

### DNA constructs and transfection

The human SDC4 was isolated and cloned into pCMV vector (Clontech Laboratories Inc.). Green fluorescent protein (GFP) was introduced to the extracellular domain of SDC4 as it was characterized elsewhere [17]. Briefly, the GFP was inserted into the extracellular domain at the position of 131 of the aminoacid sequence of human SDC4 (accession: CAG46871.1) resulted wt SDC4. The Ser179 of SDC4 was mutated to Ala or Glu obtaining Ser179Ala or Ser179Glu SDC4, respectively. Further, the PDZ binding site was deleted in the constructs of Ser179Ala or Ser179Glu creating ΔPDZ-Ser179Ala and ΔPDZ-Ser179Glu double mutants. The MCF-7 cells were transfected with the plasmids of interest by using FuGene6 or X-tremeGene HP transfection reagents (Roche Diagnostics) following the manufacturer’s instructions. For stable expression the transfected cells were selected in medium supplemented with 500 μg/ml and maintained on 200 μg/ml G418 (Cambrex).

Different cell lines were developed expressing the wt SDC4, Ser179Ala, Ser179Glu, and PDZ binding site deletion double mutants (ΔPDZ-Ser179Ala and ΔPDZ-Ser179Glu). Short hairpin RNA targeting human Tiam1 (shTiam1) was designed with the sequence of 5’-GAGACTCCTCCGTACAGTAAT and its scrambled sequence cloned into pSIREN-RetroQ plasmid (Clontech Laboratories Inc.) according to the protocol of the manufacturer. The MCF-7 cells were transiently transfected with shTiam1 construct for 48 hours prior to harvesting and processing.

### Antibodies and reagents

The next antibodies were used at appropriate dilutions: mouse monoclonal anti-GFP, Life Technologies/Zymed; polyclonal anti-SDC4: from rabbit ZMD.293, Life Technologies/Zymed; from goat D-16/sc9499 and from rabbit 5G9/sc12766, Santa Cruz Biotechnology inc.; polyclonal anti-Tiam1 C-16, Santa Cruz Biotechnology Inc.; A2484 and A2609, Sigma-Aldrich; polyclonal anti-phospho-S174-RhoGDI ab74142, Abcam Ltd; anti-RhoGDI1 (alpha): polyclonal rabbit A-20/sc-360 and monoclonal mouse B-10/sc-13120, Santa Cruz Biotechnology Inc.; polyclonal anti-Pak1 #2602, Cell Signaling Technology; anti-Rac1 monoclonal mouse #05–389, Merck Millipore; anti-Rho monoclonal mouse #05–778, Merck Millipore. HRP-conjugated goat polyclonal anti-rabbit (P0448), rabbit polyclonal anti-mouse (P0161) and rabbit polyclonal anti-goat (P0449) secondary antibodies were from DAKO Agilent Technologies.

### Microscopy and immunofluorescence staining

For immunofluorescence staining the cells were seeded on fibronectin coated glass coverslips, and fixed for 5 min with 4% paraformaldehyde or methanol. After permeabilization with 0.1% Triton X-100 for 5 min, and blocking with 1% BSA in PBS cells were incubated for 30 min at room temperature with primary antibodies diluted in 1% BSA in PBS, then were washed 3 times with PBS, incubated with secondary antibodies for 20 min at room temperature. Following the nuclear staining the coverslips were mounted with Fluorescent Mounting Medium (DAKO). Cells were studied with Zeiss Axio Imager (Zeiss) and Nikon Eclipse 600 fluorescent microscopes, or Bio-Rad MRC-1024 (Bio-Rad) confocal microscope. Images were analyzed using Adobe Photoshop^®^ v.8.

### Rac1/RhoA activation assay

Rac1 and RhoA activation was estimated by applying Rac or RhoA-activation assay kit (Merck Millipore Corporate). The Rac-kit utilizes the Cdc42/Rac1 interactive binding (CRIB) domain of Pak1 kinase and the Rho-kit based on GST-tagged RhoA binding domain of Rhotekin. The cells were treated with PKC alpha inhibitor Gö6976 (Merck Millipore Corporate) (10 nM, 1 h) or with Tiam1 inhibitor NSC23766 (Santa Cruz Biotechnology Inc.) (100 μM, 1 h), when it was stated. The active Rac1 and RhoA were detected by Western blotting using mouse anti-Rac1 or RhoA antibodies (Merck Millipore Corporate /Upstate). In control experiments cell lysates were incubated with GTP-gammaS (10 mM) or GDP (100 mM), according to the manufacturer’s instructions.

### Immunoprecipitation, Western blotting

Cells were lysed in Mg^2+^-lysis buffer (25 mM HEPES, pH 7.5, 150 mM NaCl, 1% Igepal CA-630, 5 mM MgCl_2_, 1 mM EDTA, 2% glycerol) supplemented with 1 mM Na-fluoride, 1 mM Na_3_VO_4_ and protease inhibitor cocktail (Sigma-Aldrich). The lysates were centrifuged and the supernatants were pre-cleared by addition of 5 μL of protein A/G magnetic beads slurry for 2 hours, then the supernatants were incubated with the antibody of interest overnight. 10 μl of protein A/G slurry was added for 2 hours, collected by pulse centrifugation, washed 3 times with lyses buffer, and the eluted immune complex was subjected to SDS-PAGE, followed by immunoblotting with the appropriate primary antibodies. Specific binding was detected with a secondary peroxidase-conjugated antibody (DAKO), and peroxidase activity was visualized by the ECL procedure (GE Healthcare/ Amersham).

### Statistical analysis

Data are represented as the mean from at least three independent experiments, otherwise stated. Results are expressed as means ± standard error of the mean (SEM). Differences between experimental groups were evaluated by using one-way analysis of variance (ANOVA). Values of p<0.05 were accepted as significant.

## Supporting information

S1 Fig PKC alpha did not influence the basal activity of Rac1 in MCF-7 cells. (A) PKC alpha mediated activity of Rac1 was studied without or with Gö6976 (10 nM, 60 min). Quantitation of Rac1-GTP levels was normalized to nt MCF-7 cells. These results are representatives of 3 independent experiments; data are reported as mean ±SEM (n = 3). (B) Light micrographs of MCF-7 cells indicated that there was a change in the cell morphology upon Gö6976 administration.
(JPG)

S2 Fig Uncropped images of Western blots. Black box indicated the cropped part included in the corresponding figure.
(TIF)
