# Supplementary figures and images for: The phosphomimetic mutation of syndecan-4 binds and inhibits Tiam1 modulating Rac1 activity in PDZ interaction–dependent manner
Source: PLoS One. 2017 Nov 9;12(11):e0187094. doi: 10.1371/journal.pone.0187094 (PMC5679609; doi:10.1371/journal.pone.0187094)

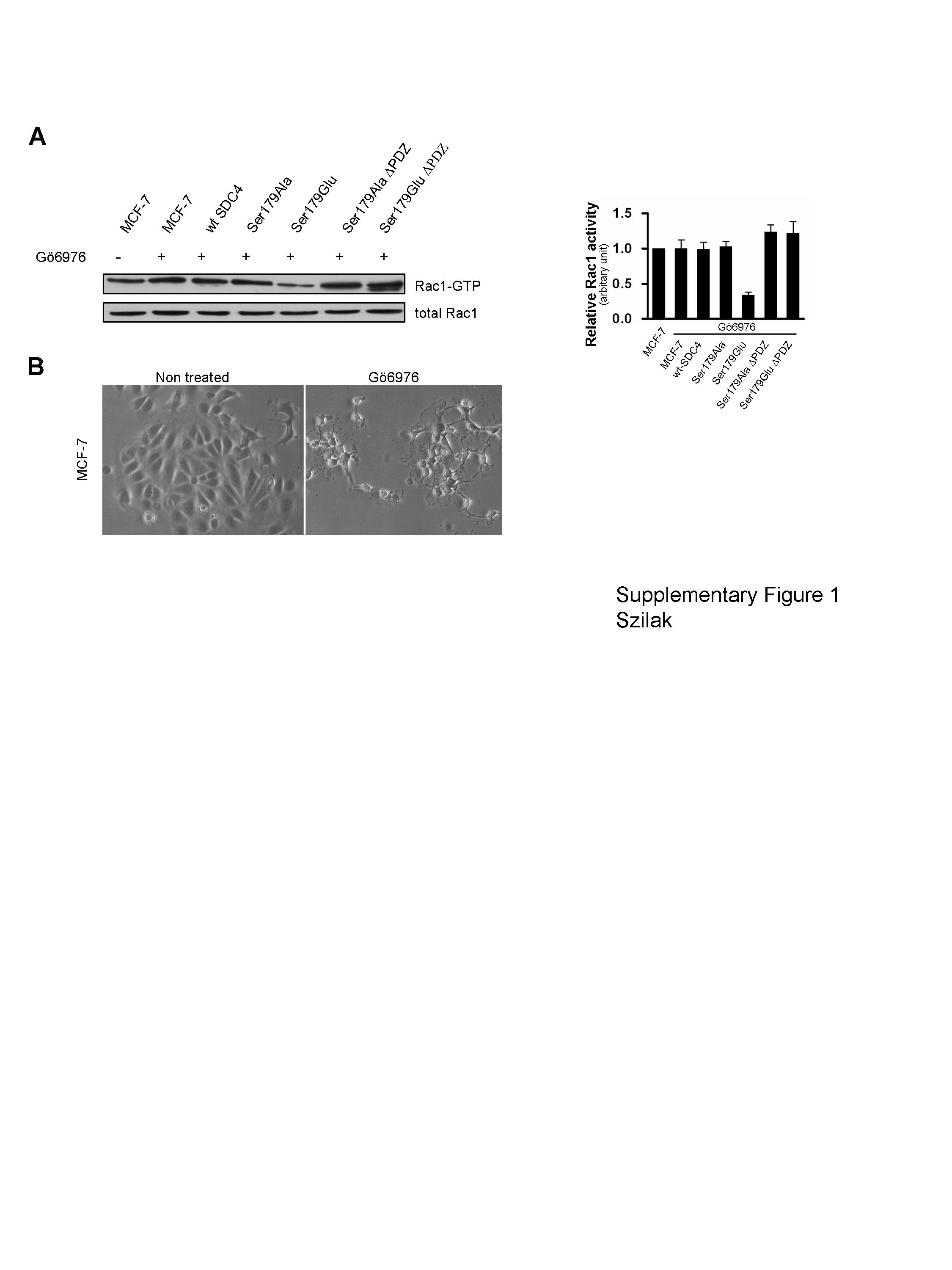

Supplement: S1 Fig — (A) PKC alpha mediated activity of Rac1 was studied without or with Gö6976 (10 nM, 60 min). Quantitation of Rac1-GTP levels was normalized to nt MCF-7 cells. These results are representatives of 3 independent experiments; data are reported as mean ±SEM (n = 3). (B) Light micrographs of MCF-7 cells indicated that there was a change in the cell morphology upon Gö6976 administration. (JPG) [file pone.0187094.s001.JPG]

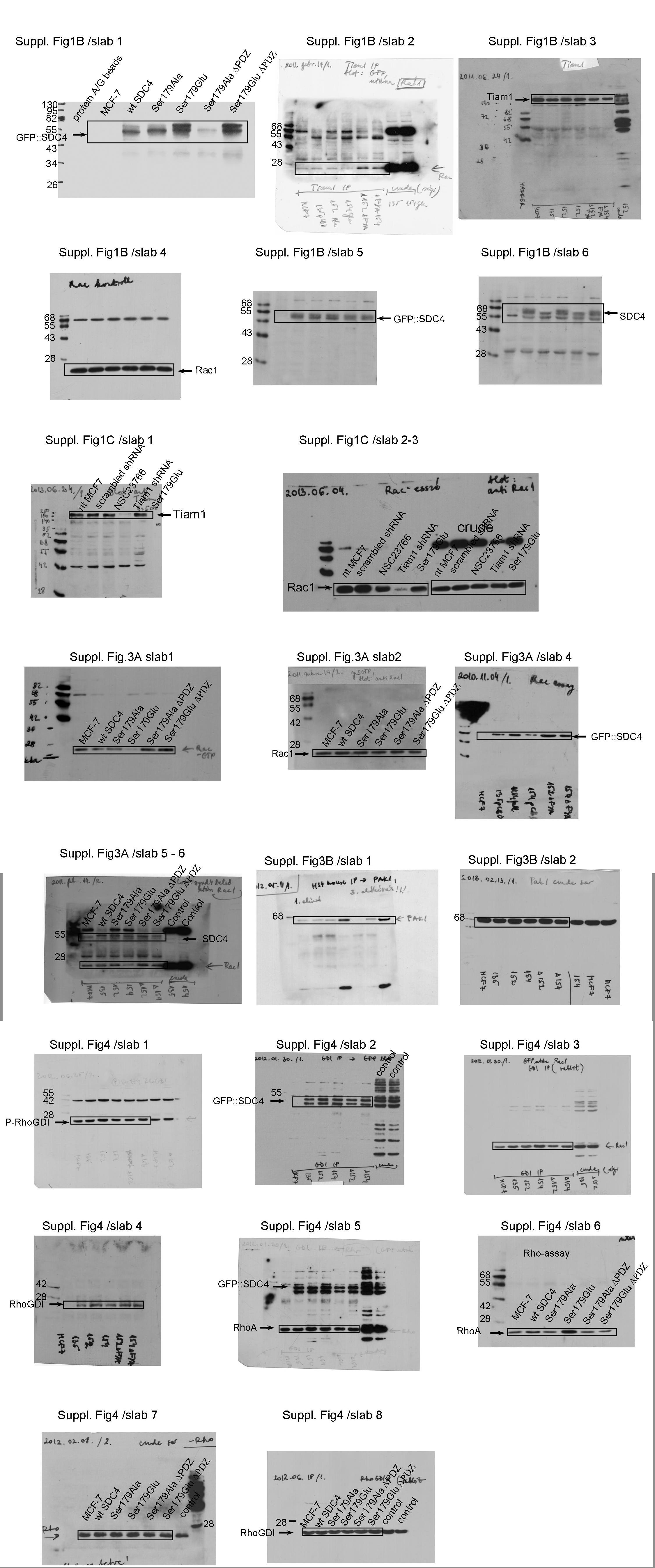

Supplement: S2 Fig — Black box indicated the cropped part included in the corresponding figure. (TIF) [file pone.0187094.s002.tif]
